# Machine learning models to predict end-stage kidney disease in chronic kidney disease stage 4

**DOI:** 10.1186/s12882-023-03424-7

**Published:** 2023-12-19

**Authors:** Kullaya Takkavatakarn, Wonsuk Oh, Ella Cheng, Girish N Nadkarni, Lili Chan

**Affiliations:** 1 Division of Nephrology, Department of Medicine, Icahn School of Medicine at Mount Sinai, New York, NY USA; 2 Division of Nephrology, Department of Medicine, King Chulalongkorn Memorial Hospital, Chulalongkorn University, Bangkok, Thailand; 3 The Charles Bronfman Institute for Personalized Medicine, Icahn School of Medicine at Mount Sinai, New York, NY USA; 4 The Cooper Union for the Advancement of Science and Art, New York, NY USA

**Keywords:** Chronic Kidney Disease, End-stage Kidney Disease, Machine learning, Artificial intelligence, Prediction model

## Abstract

**Introduction:**

End-stage kidney disease (ESKD) is associated with increased morbidity and mortality. Identifying patients with stage 4 CKD (CKD4) at risk of rapid progression to ESKD remains challenging. Accurate prediction of CKD4 progression can improve patient outcomes by improving advanced care planning and optimizing healthcare resource allocation.

**Methods:**

We obtained electronic health record data from patients with CKD4 in a large health system between January 1, 2006, and December 31, 2016. We developed and validated four models, including Least Absolute Shrinkage and Selection Operator (LASSO) regression, random forest, eXtreme Gradient Boosting (XGBoost), and artificial neural network (ANN), to predict ESKD at 3 years. We utilized area under the receiver operating characteristic curve (AUROC) to evaluate model performances and utilized Shapley additive explanation (SHAP) values and plots to define feature dependence of the best performance model.

**Results:**

We included 3,160 patients with CKD4. ESKD was observed in 538 patients (21%). All approaches had similar AUROCs; ANN yielded the highest AUROC (0.77; 95%CI 0.75 to 0.79) and LASSO regression (0.77; 95%CI 0.75 to 0.79), followed by random forest (0.76; 95% CI 0.74 to 0.79), and XGBoost (0.76; 95% CI 0.74 to 0.78).

**Conclusions:**

We developed and validated several models for near-term prediction of kidney failure in CKD4. ANN, random forest, and XGBoost demonstrated similar predictive performances. Using this suite of models, interventions can be customized based on risk, and population health and resources appropriately allocated.

## Introduction

Chronic kidney disease (CKD) is a major global public health problem that affects more than 850 million individuals worldwide [1]. In the United States, approximately 15% of the population, or 37 million people, suffer from CKD [2] and more than 130,000 CKD patients were newly diagnosed with end-stage kidney disease (ESKD) [3]. In advanced CKD, including CKD stage 4, care goals focus on slowing CKD progression and preparing for renal replacement therapy (RRT), such as dialysis modality selection, vascular access placement, and pre-emptive transplantation. According to the 2019 United States Renal Data System (USRDS), approximately 30% of incident ESKD patients did not receive nephrology care prior to being diagnosed with ESKD [3] which results in increased unplanned dialysis and early mortality after dialysis initiation [4]. Clinical decisions for CKD stage 4 are challenging in current practice due to the heterogeneity of kidney diseases and the variability of disease progression rates. Accurate prediction of the risk of kidney failure could lead to better overall CKD stage 4 management by improving individual advanced CKD care outcomes through information sharing for patients’ decision-making and matching therapy risks or side effects to the risk of disease progression. In addition, reliable prediction models enhance the efficacy of the health system by optimizing resource allocation and matching individual risk.

The development of clinical medicine’s digitization and the widespread availability of electronic health records (EHR) have generated large-scale real-world clinical data which can be used for developing clinical decision systems. Machine learning (ML) represents more sophisticated mathematical functions than traditional statistics and typically yields superior performance when predicting outcomes determined by a large number of variables with nonlinear and complex interactions [5, 6]. To date, only a few studies have developed ML prediction models for CKD progression to kidney failure, and the results have been contradictory. Whether ML predicts CKD progression better than traditional statistical analysis remains unclear.

In this study, we aimed to determine if ML models could be used to predict the progression to ESKD in patients with CKD stage 4. We hypothesized that incorporating several baseline clinical parameters in ML models would enable accurate identification of patients at high risk of developing ESKD within three years after CKD stage 4 diagnosis.

## Methods

### Study population

We included all patients who were ≥ 18 years old and had two outpatient measurements of eGFR between 15 and 30 mL/min/1.73m^2^ with at least a 3-month interval from January 1, 2006 to December 31, 2016. We calculated the estimated glomerular filtration rate (eGFR) using serum creatinine with the race free 2021 CKD Epidemiology Collaboration equation (CKD-EPI) Eq. [7]. The index date was recorded as the second eGFR measurement. Patients with an eGFR of less than 15 mL/min/1.73 m^2^ before the index date or who had a history of RRT in any form, including hemodialysis, peritoneal dialysis, and kidney transplantation, were excluded.

### Data source

We used EHR data from Mount Sinai Hospital (MSH), Mount Sinai Queens, Mount Sinai West, Mount Sinai Morningside, and Mount Sinai Brooklyn, which are all part of the Mount Sinai Health System (MSHS). The clinical data were extracted from Mount Sinai’s Epic Caboodle database and other ancillary systems, transformed into the OMOP Common Data Model (CDM) format and loaded into the Mount Sinai Data Warehouse (MSDW) database.

### Feature selection

We collected information on patient demographics (age, sex, race, and ethnicity), comorbidities derived from the International Classification of Diseases 10 (ICD-10) codes according to the Elixhauser comorbidity index, clinical parameters and vital sign measurements (body mass index (BMI), systolic and diastolic blood pressure, heart rate, respiratory rate, and body temperature), and laboratory results. Variables with more than 30% missing values were not included in the analysis. We excluded patients with > 30% missing data across the remaining features. All other missing data were imputed using predictive mean matching techniques with five imputations based on the Multivariate Imputation via Chained Equations (MICE) function in R version 4.2.2.

### Outcomes

The outcome was ESKD defined by eGFR < 15 ml/min/1.73m^2^ at least two measurements over a period of more than 3 months or the initiation of RRT (dialysis or kidney transplantation) within 3 years after CKD stage 4 diagnosis.

### Model development and selection

The model was trained to predict a binary classification problem with the objective of generating the probability of an outcome based on the features provided. Several algorithms were employed in this study, including logistic regression with L1 regularization (LASSO regression), random forest, eXtreme Gradient Boosting (XGBoost), and feed-forward artificial neural network (ANN, a deep learning model).

Random forest and XGBoost are both decision tree ensemble algorithms. While random forest works on bagging, XGBoost utilizes gradient descent-boosting. Random forest trains each tree independently and selects the average prediction values of the individual trees for regression problems and the maximum vote for classification problems. [8]. In contrast, XGBoost processes the data sequentially, with each newly fitted tree dependent on the previous one in order to minimize the error [9]. ANN is produced by assembling multiple layers with linear or nonlinear activation functions. A multilayer feed-forward neural network with backpropagation and stochastic gradient descent was used to classify the data.

Before modeling, all categorical variables with more than two factors were one-hot encoded (turning categorical variable factors into a separate binary variable). Then, all the models were trained and validated using a five-fold cross-validation approach. The dataset was randomly divided into five folds, 80% for training and 20% for validation. The cross-validation process is then repeated five times. Each iteration used a different stratified fold for model evaluation, and the remaining folds were used for model training.

### Hyperparameter tuning

Grid search was performed to obtain the best combination of hyperparameters using cross-validation methods for the random forest, XGBoost, and ANN. For LASSO regression, cross-validation and the value of λ that yields the minimum mean cross-validated error were employed. The final hyperparameters for each model are listed in Supplementary Table 1.

### Model evaluation

Model performance was evaluated using the area under the receiver operating characteristic (AUROC) curve and the area under the precision-recall curve (AUPRC) to account for the skewed distribution of the outcome, a minority of patients within the cohort developed ESKD. The baseline of AUPRC is determined by the fraction of positive cases where an AUPRC above this fraction is regarded as a better than chance. We also evaluated the accuracy and precision of the models. Due to the different classification models obtained for each hyperparameter combination and during each training fold, the model with the highest AUROC on the validation set was selected as the final model and was trained on all training data. 95% confidence intervals were generated through 1000 bootstrap iterations with a unique random seed.

Model calibration was assessed using the Brier score and reliability diagram. The Brier score is defined as the mean squared difference between the observed and predicted outcomes and ranges from 0 to 1.00, with 0 representing the best possible calibration. [10] Reliability diagrams were used to plot the mean risk score relative to the observed outcome rate for a given quintile of the predicted risk. The clinical value of the model was evaluated using decision curve analysis (DCA). Net benefit was computed by subtracting the proportion of false positives from the proportion of true positives in all patients, weighing relative harm driven by the false positive. [11].

### Statistical analysis

Categorical data are described as numbers and percentages. Continuous data are summarized as mean ± standard deviation (sd) for normally distributed variables or median (interquartile range; IQR) for non-normally distributed variables. We used Student’s T test for normally distributed continuous variables, Kruskal-Wallis for non-normally distributed continuous variables, and χ2 for categorical variables. A p < 0.05 was considered statistically significant. All analyses were performed using R, version 4.2.2 (RStudio, Inc., Boston, MA, USA). We used the “glmnet” package (version 4.1-8) for LASSO regression, “randomForest” package (version 4.7–1.1) for random forest, “xgboost” package (version 1.6-1) for XGBoost, and “caret” (version 6.0–94) and “keras” packages (version 2.9.0) for ANN. The “mice” package (version 3.14.0) was utilized to impute missing data.

## Results

### Baseline characteristics

We included 3,160 patients for analysis. The mean age of the cohort was 69 ± 11 years with a mean eGFR of 25 ± 4 ml/min/1.73m^2^. Of the patients, 53% were female, 48% had diabetes mellitus, 75% had hypertension, and 45% had a history of cardiovascular diseases (including previous myocardial infarction, congestive heart failure, stroke, and peripheral vascular disease). The baseline characteristics of these patients are shown in Table 1.

**Fig. 1:**
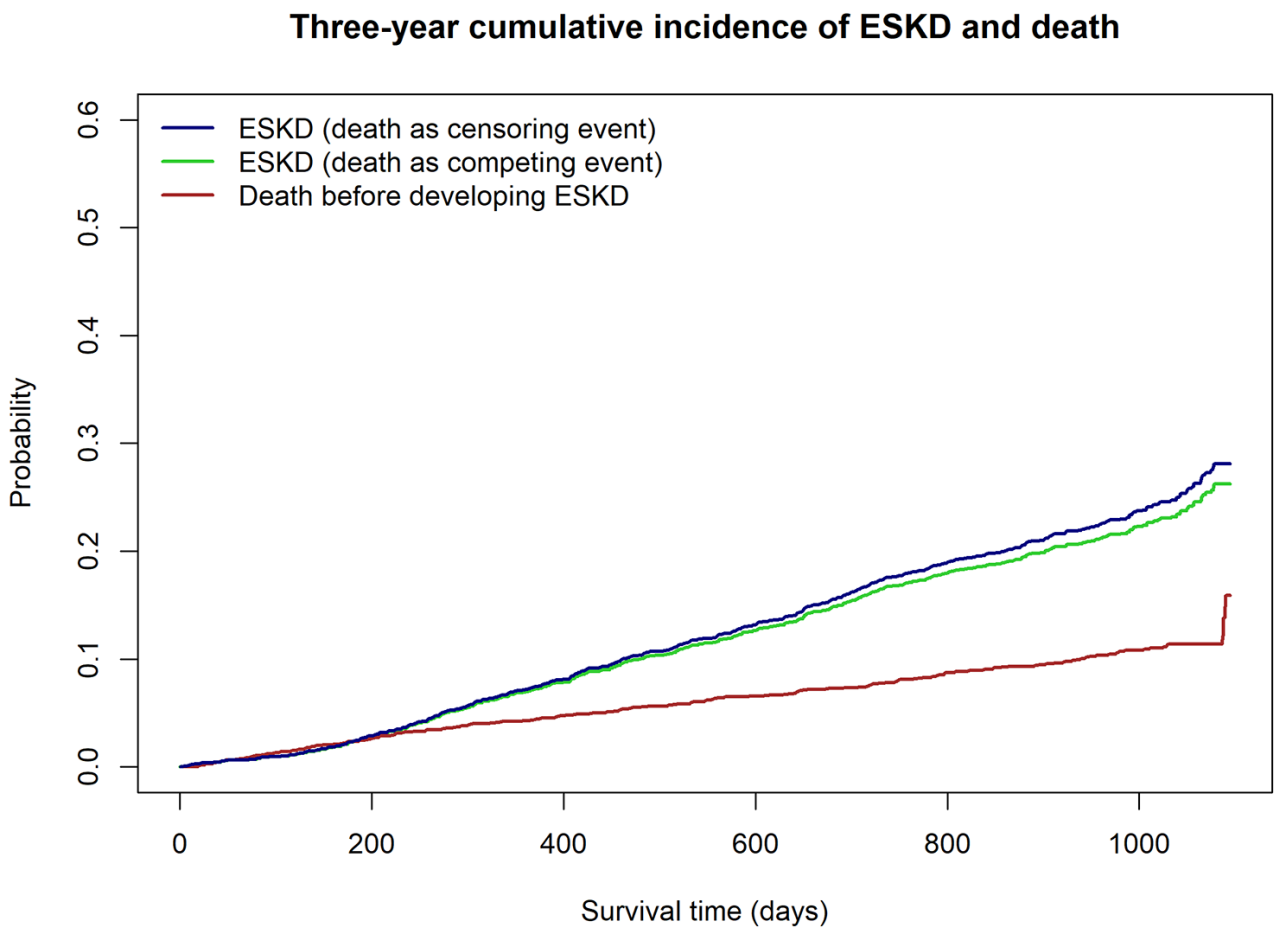
Three-year cumulative incidence of ESKD and death

During 3 years of follow-up, there were 538 patients (21%) who developed ESKD and 291 patients (9.2%) who died before developing ESKD. Figure 1 shows the crude risks in the cohort estimated by the Kaplan-Meier and the competing risk analyses. The cumulative incidence of ESKD estimated by Kaplan-Meier analysis was comparable to the cumulative incidence estimate that accounts for the competing risk of death.

**Table 1:** Baseline characteristics of the patients

Characteristics	Missing data (%)	Progressed to ESKD (n = 538)	Non-progressed to ESKD (n = 2,622)	p-value
Demographic				
Age (years)	0	62 ± 14	70 ± 10	< 0.0001
Female	0	267 (50)	1,428 (55)	
Race	0			< 0.0001
Black		86 (16)	315 (12)	
Hispanic		57 (10.6)	263 (10)	
White		156 (29)	1,055 (40.2)	
Others		239 (44.0)	989 (37.7)	
BMI	20	29.5 [25, 34.5]	28 [25, 33]	0.002
**Comorbidities**				
Hypertension	0	404 (75)	1,975 (75)	0.90
Diabetes mellitus	0	277 (52)	1.245 (47)	0.09
Congestive heart failure	0	114 (21)	587 (22)	0.54
Myocardial infarction	0	132 (25)	792 (30)	0.008
Stroke or TIA	0	32 (6)	179 (7)	0.45
Peripheral arterial disease	0	93 (17)	484 (19)	0.52
Cardiovascular disease	0	238 (44)	1,209 (46)	0.42
Arrhythmia	0	74 (13)	552 (21)	< 0.0001
Valvular heart disease	0	43 (8)	282 (11)	0.01
Pulmonary embolism	0	7 (1)	47 (2)	0.42
Pulmonary hypertension	0	17 (4)	90 (4)	0.75
Liver disease	0	62 (12)	322 (12)	0.46
HIV infection	0	0	1 (0.1)	0.65
Solid malignancy	0	48 (9)	492 (19)	< 0.0001
Lymphoma	0	18 (3)	98 (4)	0.80
Anemia	0	44 (8)	222 (9)	0.84
Peptic ulcer	0	7 (1)	59 (2)	0.16
Connective tissue diseases	0	20 (4)	100 (4)	0.92
Psychosis	0	6 (1)	36 (1)	0.63
Depression	0	55 (10)	343 (13)	0.07
**RAAS inhibitor**	0	206 (38)	1,062 (41)	0.34
**Vital signs**				
Systolic blood pressure	0	138 ± 26	132 ± 22	< 0.0001
Diastolic blood pressure	0	75 ± 13	73 ± 12	< 0.0001
Heart rate	6	76 ± 14	75 ± 14	0.13
Respiratory rate	15	17 ± 3	17 ± 3	0.29
**Laboratory values**				
BUN	0	47 ± 17	44 ± 17	0.0009
Creatinine	0	2.7 ± 0.6	2.3 ± 0.5	< 0.0001
eGFR	0	23.11 ± 4.46	25.78 ± 3.66	< 0.0001
Hemoglobin	3	11.1 ± 1.7	11.5 ± 1.8	< 0.0001
Hematocrit	3	33 ± 5	35 ± 5	< 0.0001
Sodium	0	140 ± 3	139 ± 4	0.0001
Potassium	0	4.7 ± 1.4	4.7 ± 0.7	0.36
Bicarbonate	0	23 ± 4	24 ± 4	< 0.0001
Calcium	1	9.2 ± 0.7	9.4 ± 0.7	< 0.0001
Phosphate	0	3.9 ± 0.03	3.8 ± 0.7	0.0005
Albumin	3	3.7 ± 0.6	3.9 ± 1.4	0.0009
SGPT	6	17 [13, 25]	17 [13, 25]	0.39
Alkaline phosphatase	7	90 [68,119]	85 [66, 108]	0.02
Total bilirubin	7	0.3 [0.2, 0.5]	0.4 [0.3, 0.6]	0.04
Total cholesterol	25	170 [141, 207]	162 [137, 193]	< 0.0001
LDL	28	91 [69, 1167]	83 [64, 106]	< 0.0001
HDL	28	51 [38, 61]	48 [39, 60]	0.76

### Model performance

**Fig. 2:**
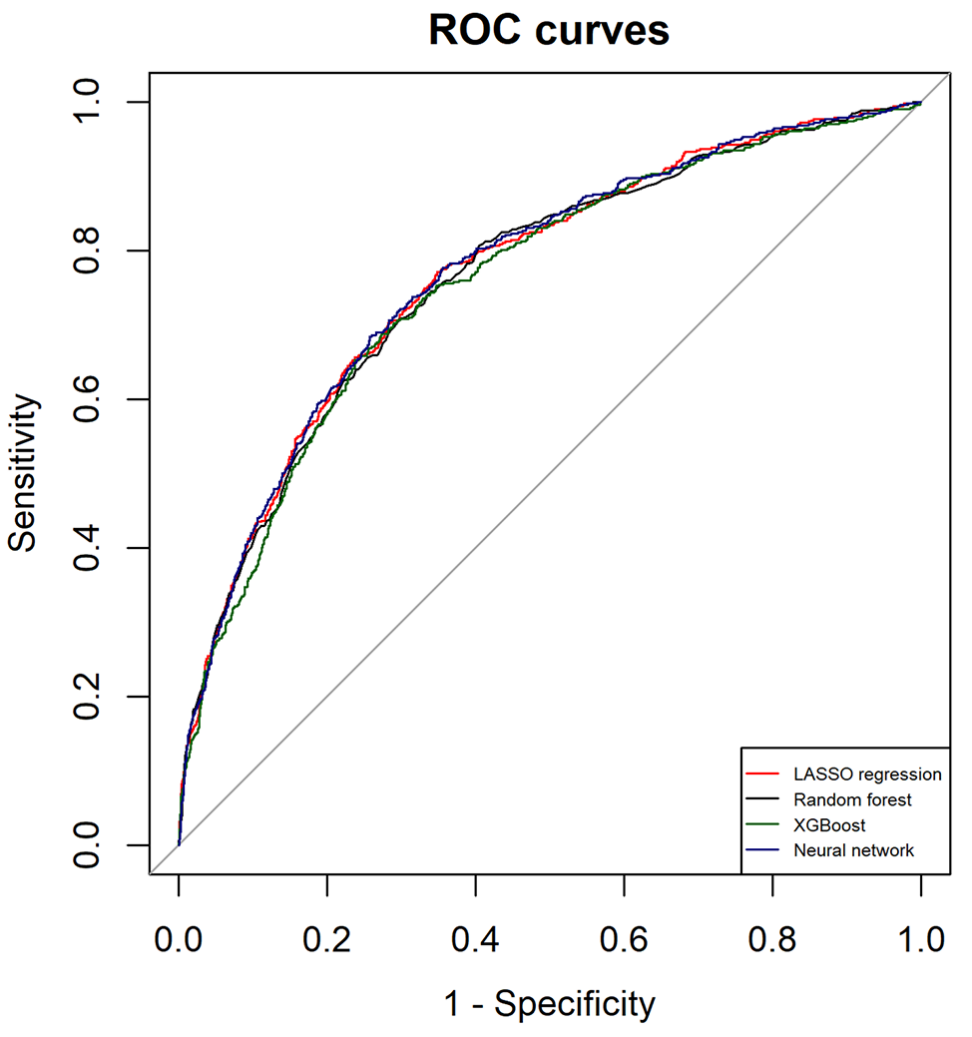
Receiver-operating characteristic (ROC) curves of each model

**Fig. 3:**
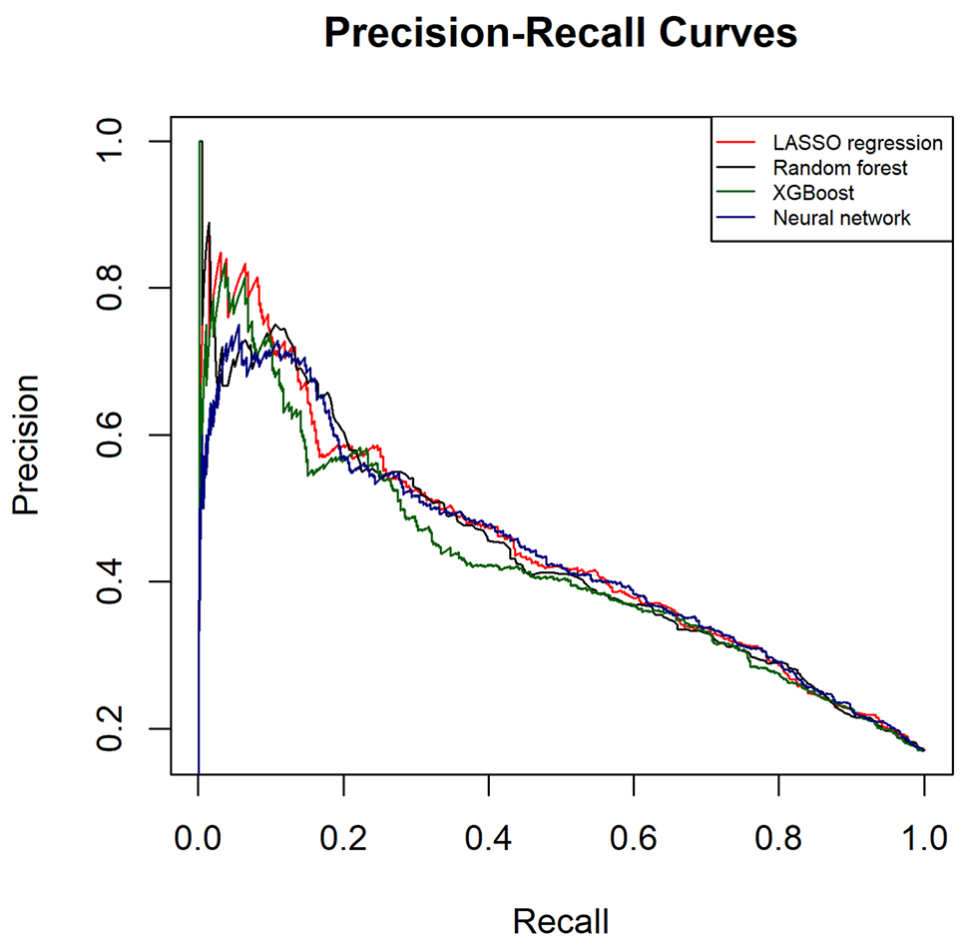
Precision-Recall curves of each model

**Fig. 4:**
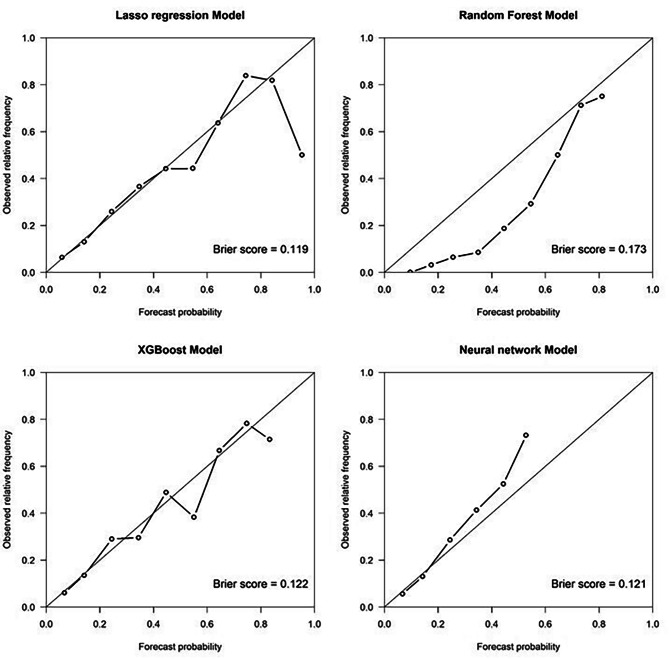
Reliability diagram of all models

**Fig. 5:**
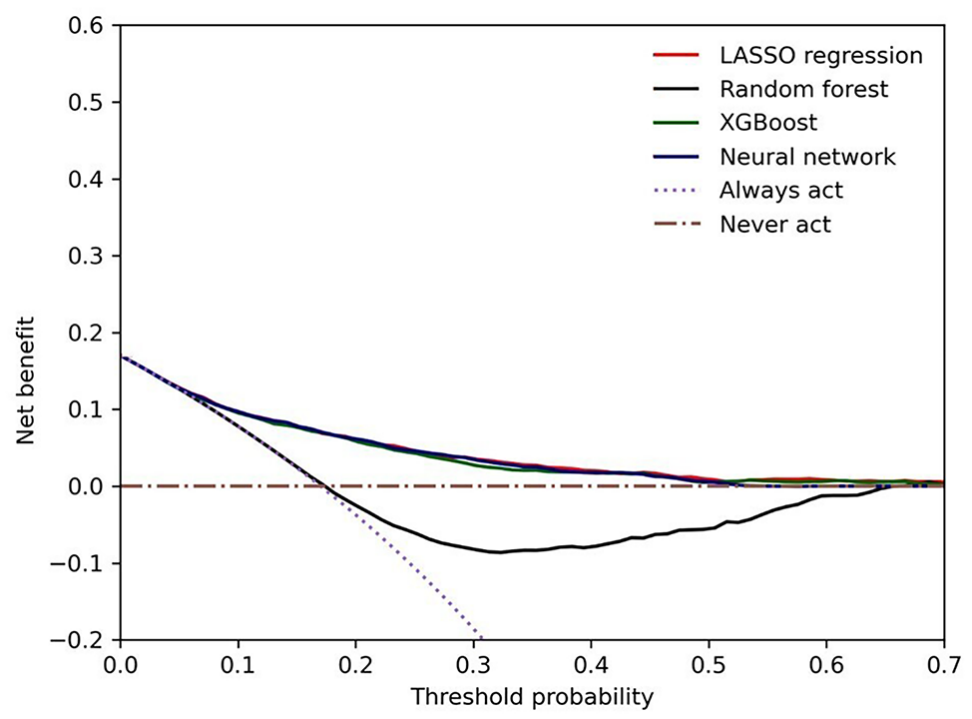
Decision curve analysis of all models

The performance metrics of all models are shown in Table 2. All models had AUROC values greater than 0.76 and had AUPRC values higher than a fraction of positive cases (0.2). ANN and LASSO regression showed the highest AUROC (0.77; 95%CI 0.75 to 0.79), LASSO regression had the highest AUPRC (0.45; 95%CI 0.40 to 0.49) while ANN yielded the highest precision (73%). Figures 2 and 3 illustrate the ROC and PRC curves for each model, respectively. All models exhibited good calibration, with Brier scores ranging from 0.12 to 0.17. The reliability diagrams in Fig. 4 display a strong agreement between actual observations and model predictions. Figure 5 shows the results of DCA for all models. The net benefit was generally great for the LASSO regression, XGBoost, and ANN models, while the random forest had the lowest clinical utility. Model performance was compared in each eGFR, sex, race, comorbidity, and laboratory value, as shown in Table 3.

**Table 2:** Model performance for prediction of ESKD at 3 years

Model	Model performance metric			
	Accuracy	Precision	AUROC	AUPRC
**LASSO**	0.83 (0.82 to 0.85)	0.58 (0.51 to 0.66)	**0.77** **(0.75 to 0.79)**	**0.45** **(0.40 to 0.49)**
**Random Forest**	0.82 (0.81 to 0.83)	0.38 (0.35 to 0.42)	0.76 (0.74 to 0.79)	0.44 (0.39 to 0.48)
**XGBoost**	**0.84** **(0.83 to 0.85)**	0.38 (0.35 to 0.42)	0.76 (0.74 to 0.78)	0.43 (0.38 to 0.47)
**Neural network**	0.83 (0.82 to 0.85)	**0.73** **(0.60 to 0.87)**	**0.77** **(0.75 to 0.79)**	0.44 (0.39 to 0.48)

**Table 3:** Subgroup analyses for prediction of ESKD at 3 years

Subgroup	AUROC			
	LASSO regression	Random forest	XGBoost	Neural network
eGFR				
15–20 mL/min/1.73m2 (n = 413)	0.69 (0.63 to 0.74)	0.69 (0.64 to 0.75)	0.65 (0.63 to 0.74)	0.70 (0.65 to 0.75)
20–29 mL/min/1.7m2 (n = 1,747)	0.74 (0.72 to 0.77)	0.74 (0.71 to 0.76)	0.73 (0.71 to 0.76)	0.75 (0.72 to 0.77)
**Sex**				
Female (n = 1,695)	0.79 (0.76 to 0.82)	0.78 (0.75 to 0.81)	0.77 (0.74 to 0.81)	0.79 (0.76 to 0.82)
Male (n = 1,465)	0.75 (0.71 to 0.78)	0.74 (0.71 to 0.77)	0.74 (0.71 to 0.78)	0.75 (0.72 to 0.78)
**Race**				
Black (n = 401)	0.74 (0.68 to 0.80)	0.75 (0.69 to 0.81)	0.75 (0.66 to 0.79)	0.74 (0.68 to 0.80)
Hispanic (n = 320)	0.73 (0.66 to 0.80)	0.71 (0.64 to 0.79)	0.71 (0.65 to 0.79)	0.73 (0.66 to 0.80)
White (n = 1,211)	0.77 (0.73 to 0.81)	0.78 (0.74 to 0.81)	0.78 (0.72 to 0.81)	0.77 (0.73 to 0.81)
Others (n = 1,228)	0.78 (0.74 to 0.81)	0.76 (0.73 to 0.80)	0.76 (0.74 to 0.81)	0.78 (0.75 to 0.81)
**Comorbidity**				
BMI ≥ 25 kg/m2 (n = 1,424)	0.78 (0.75 to 0.80)	0.78 (0.75 to 0.80)	0.77 (0.75 to 0.80)	0.78 (0.76 to 0.81)
Diabetes (n = 1,522)	0.76 (0.73 to 0.79)	0.71 (0.73 to 0.79)	0.76 (0.72 to 0.79)	0.76 (0.72 to 0.79)
Cardiovascular disease	0.75	0.77	0.76	0.77
(n = 1,447)	(0.72 to 0.79)	(0.73 to 0.80)	(0.72 to 0.79)	(0.73 to 0.80)
**Laboratory value**				
K > 5.5 mEq/L	0.82	0.82	0.80	0.82
(n = 282)	(0.75 to 0.89)	(0.75 to 0.89)	(0.74 to 0.88)	(0.75 to 0.89)
Phosphate > 5 mg/dL	0.73	0.71	0.68	0.73
(n = 114)	(0.64 to 0.81)	(0.61 to 0.80)	(0.62 to 0.80)	(0.64 to 0.82)
Serum albumin < 3.0	0.80	0.79	0.78	0.78
(n = 110)	(0.73 to 0.86)	(0.72 to 0.86)	(0.70 to 0.85)	(0.71 to 0.85)

### Feature importance

**Fig. 6:**
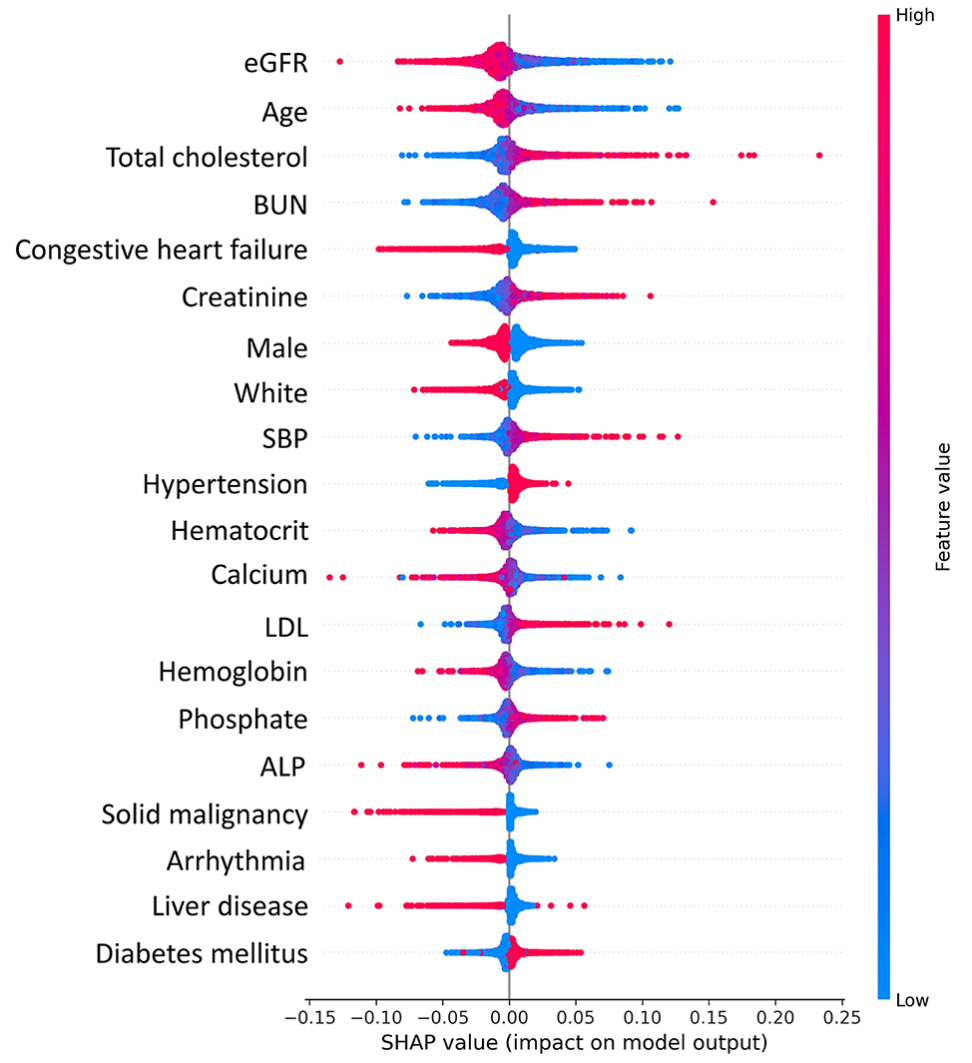
The Shapley additive explanations (SHAP) plot for the neural network model demonstrates the importance of relative features. Each plot is comprised of individual points from the training dataset, with higher feature values represented by darker red and lower values by greater blue

To identify the features that had the most impact on the best prediction model with the highest AUROC, we calculated Shapley additive explanations (SHAP) scores for all patients and generated summary plots of the top 20 features in the ANN prediction model (Fig. 6). This plot illustrates how high and low the values of the testing dataset features were relative to SHAP values based on their importance—the risk of developing ESKD increases as the SHAP value of a feature increases. According to the prediction model, eGFR at baseline and age, were the most influential model predictors. Other clinically significant features included total cholesterol, BUN, history of congestive heart failure, serum creatinine, systolic blood pressure, hypertension, and hematocrit.

## Discussion

In this retrospective study, we developed and cross-validated several predictive models for the risk ESKD in patients with CKD4 over a 3-year follow-up period. EHR clinical variables were comprehensively incorporated into the models, including demographic, comorbidity, vital signs, and baseline laboratory data. LASSO regression analysis was used as the traditional statistical model, whereas the ML model comprised random forest, XGBoost, and ANN. The outcomes demonstrated that traditional, tree ensemble, and ANN algorithms provided comparable performance.

Although ML models have been demonstrated to outperform traditional statistical models in several tasks in nephrology, such as the prediction of perioperative acute kidney injury [12, 13], short-term mortality after dialysis [14, 15], and time to allograft losses [16]. Conversely, several recent studies that developed ML prediction models for the progression of CKD to kidney failures yielded conflicting outcomes. Some investigations revealed the superior performance of ML models compared to other methods [17–19]; however, other studies supported our findings by displaying comparable or even inferior performance of ML models compared to traditional regression models. Bai et al. reported the equivalent 5-year ESKD predictability of random forest, logistic regression, and the Kidney Failure Risk Equation in 748 CKD patients [20]. Similarly, Xiao et al. assessed the progression of CKD using urine protein prediction in 551 CKD patients and revealed insignificant differences in predicting performance between ML and regression models [21]. Apart from the studies with relatively small datasets, a large cohort using data from 8,500 CKD patients to predict RRT within 12 months demonstrated that logistic regression provided the highest AUROC compared with other ML models [22].

Theoretically, ML models can improve the predictive ability achieved by regression models when given a sufficient sample size and number of predictor variables. ML models enable the detection of nonlinear and complex interactions and provide more accurate predictions. However, in the present study, the performance of ML and the traditional model was comparable. We believe that given the relative short transition time for CKD 4 to 5 may be contributing to the lack of improvement in performance of ML models compared to LASSO. Risk models generally assume that disease progresses in a certain direction, called trajectory. Generalized linear models, the first-hand model in clinical research, can capture the disease trajectory based on the linearity assumption. However, two sources of non-linearity may lead to the underfitting of linear models for risk modeling. First, multiple trajectories can exist due to the heterogeneity of the underlying biological mechanism and patients’ environmental differences. Second, not every feature has a linear and monotonical relationship with the disease progression; some present a convex, concave, exponential, or logarithmic relationship with outcomes. ML models can address these non-linear interactions naturally, while complex models are potentially prone to overfitting. Thus, conditions for the success of linear and ML models are exclusive. Unfortunately, patients with CKD 4 to 5 have a considerably short transition time, so heterogeneity and non-additive effect are likely negligible for the differentiation of the onset of ESKD.

Among the clinical variables, age and baseline eGFR were determined to be the most predictive features, followed by CKD-associated biochemical and physiological disturbances such as calcium, phosphate, hemoglobin, and systolic blood pressure, which is consistent with previous studies [18, 23]. Apart from established predictors, total cholesterol, LDL, and alkaline phosphatase were found to contribute to the prediction of progression from CKD 4 to ESKD. These findings from a data-driven approach provide clinicians with important information about additional factors to monitor in patients with CKD 4.

Our study has important clinical implications. To the best of our knowledge, most of the previous ML prediction models for CKD progression have been studied in patients with moderate CKD (mean eGFR range of 45 to 66 mL/min/1.73m2) [19, 20, 23]. While our study developed an ML model and validated it in patients with an eGFR of less than 30 mL/min/1.73m^2^, who are concerned with slowing CKD progression and preparing for RRT. Previously, Cheng et al. developed models using the temporal abstraction technique and data mining methods, including classification and regression tree, and adaptive boosting (AdaBoost), to predict CKD progression over a relatively shorter period at 6 months in 463 CKD stage 4 patients. The models achieved an accuracy of 0.66 and an AUROC of 0.71. [24]. From a clinical perspective, our models can identify patients at high risk of progression to ESKD in the following three years. Patients with CKD 4, particularly, those with a high risk of ESKD progression, should be considered for referral to multidisciplinary, comprehensive clinical management by nephrology specialists. In addition, the use of renin-angiotensin-aldosterone (RAAS) inhibitors and recently approved drugs, such as SGLT2 inhibitors, are widely recognized as one of the most effective methods for delaying the progression of the disease in early CKD [25–27]. However, it remains unclear if these interventions are suitable for advanced CKD. Furthermore, risk prediction of ESKD may provide appropriate time for advising and educating patients about a pre-emptive kidney transplant, preparing for vascular access placement, and avoiding emergent initiation of hemodialysis using a catheter [28, 29].

Our study has some limitations. First, while we used data from five different hospitals, they were all part of Mount Sinai and we have not tested the generalizability of these models on external data. Secondly, proteinuria, a known risk factor of CKD progression, was missing in more than 42% of the cohort and was excluded from the analysis. Although this is representative of current practice, and we aimed to develop prediction models using real-world EHR data, a further study on the more widespread availability of proteinuria may enhance the performance of the models and should be addressed in future studies. Lastly, our models did not take into account the competing risk of death, which plays an essential role in risk assessment for patients with advanced CKD who are older and frail. A previous study revealed that the 5-year Kidney Failure Risk Equation (KFRE), one of the existing prediction models used in clinical practice, overestimated risk by 10–18% due to the competing risk of death [30]. However, in our cohort, the mortality rate of CKD 4 patients is much lower than that reported in previous studies [31]. Consequently, the conventional and competing risk analyses yielded similar results (Fig. 1). Further studies should prioritize the external validation of these models and explore the utilization of competing risk models that account for mortality, particularly in cohorts characterized by higher mortality rates among CKD stage 4 patients. Furthermore, exploring the effectiveness of alternative deep learning models is warranted. Finally, it is crucial to investigate the impact of implementing these models in clinical management and assess outcomes in clinical trials.

## Conclusion

We present new ESKD prediction models for patients with advanced CKD based on EHR clinical data. Random forest, XGBoost, and ANN demonstrated comparable predictability to the LASSO regression models in this study. With these models, therapeutic interventions can be customized based on risk for CKD 4 patients, and strategies for patient requirements and healthcare system resources can be appropriately planned.

## Data Availability

The data underlying this article will be shared on reasonable request to the corresponding author. Our institution has a data use committee and due processes requiring transfer of data external to our institution.

## Declarations

The study was performed in accordance with the Declaration of Helsinki and Ethical Guidelines for Medical and Health Research Involving Human Subjects. The study was approved by the Mount Sinai Institutional Review Board (STUDY-20-00338) which allowed for analysis of patient-level data with a waiver of informed consent.
